# Rescue of infectious Arumowot virus from cloned cDNA: Posttranslational degradation of Arumowot virus NSs protein in human cells

**DOI:** 10.1371/journal.pntd.0007904

**Published:** 2019-11-21

**Authors:** Hoai J. Hallam, Nandadeva Lokugamage, Tetsuro Ikegami

**Affiliations:** 1 Department of Pathology, The University of Texas Medical Branch at Galveston, Galveston, Texas, United States of America; 2 Sealy Institute for Vaccine Sciences, The University of Texas Medical Branch at Galveston, Galveston, Texas, United States of America; 3 Center for Biodefense and Emerging Infectious Diseases, The University of Texas Medical Branch at Galveston, Galveston, Texas, United States of America; Faculty of Science, Ain Shams University (ASU), EGYPT

## Abstract

Rift Valley fever (RVF) is a mosquito-borne zoonotic disease endemic to Africa and the Middle East, affecting both humans and ruminants. There are no licensed vaccines or antivirals available for humans, whereas research using RVF virus (RVFV) is strictly regulated in many countries with safety concerns. Nonpathogenic Arumowot virus (AMTV), a mosquito-borne phlebovirus in Africa, is likely useful for the screening of broad-acting antiviral candidates for phleboviruses including RVFV, as well as a potential vaccine vector for RVF. In this study, we aimed to generate T7 RNA polymerase-driven reverse genetics system for AMTV. We hypothesized that recombinant AMTV (rAMTV) is viable, and AMTV NSs protein is dispensable for efficient replication of rAMTV in type-I interferon (IFN)-incompetent cells, whereas AMTV NSs proteins support robust viral replication in type-I IFN-competent cells. The study demonstrated the rescue of rAMTV and that lacking the NSs gene (rAMTVΔNSs), that expressing green fluorescent protein (GFP) (rAMTV-GFP) or that expressing *Renilla* luciferase (rAMTV-rLuc) from cloned cDNA. The rAMTV-rLuc and the RVFV rMP12-rLuc showed a similar susceptibility to favipiravir or ribavirin. Interestingly, neither of rAMTV nor rAMTVΔNSs replicated efficiently in human MRC-5 or A549 cells, regardless of the presence of NSs gene. Little accumulation of AMTV NSs protein occurred in those cells, which was restored via treatment with proteasomal inhibitor MG132. In murine MEF or Hepa1-6 cells, rAMTV, but not rAMTVΔNSs, replicated efficiently, with an inhibition of IFN-β gene upregulation. This study showed an establishment of the first reverse genetics for AMTV, a lack of stability of AMTV NSs proteins in human cells, and an IFN-β gene antagonist function of AMTV NSs proteins in murine cells. The AMTV can be a nonpathogenic surrogate model for studying phleboviruses including RVFV.

## Author summary

Rift Valley fever virus (RVFV) is a mosquito-borne phlebovirus endemic to Africa and the Middle East, causing devastating outbreaks affecting both humans and animals. The reverse genetics system for RVFV has contributed to the virology, vaccinology, and antiviral screening for RVFV. In this study, we generated the first reverse genetics system for a mosquito-borne nonpathogenic phlebovirus (Arumowot virus; AMTV) endemic to Africa, which is phylogenetically related to RVFV. The generation of recombinant AMTV supports the screening of broad-acting antivirals and vaccine development for RVF. The nonstructural NSs protein is known as a major virulence factor for RVF, yet this study revealed that AMTV NSs protein was rapidly degraded in human cells via cellular proteasomes. In contrast, AMTV NSs protein functioned as an antagonist of interferon-β gene upregulation in murine cells. The AMTV can be a nonpathogenic surrogate model for studying phleboviruses including RVFV.

## Introduction

Rift Valley fever (RVF) is one of the most important zoonotic viral diseases for public health, which is classified as Category A Priority Pathogen by the National Institute of Allergy and Infectious Diseases in the United States (U.S.) and the Blueprint priority disease by the World Health Organization [1, 2]. RVF had been endemic to sub-Saharan Africa, and has spread into Egypt, Madagascar, the Comoros, Saudi Arabia, and Yemen [3]. RVF is characterized by a high rate of abortions and fetal malformations in pregnant ewes, goats or cattle, and high mortality of newborn lambs or goat kids due to acute liver necrosis [4]. In humans, most patients suffer from self-limiting febrile illness, whereas some patients develop hemorrhagic fever, encephalitis, or vison loss [5]. Despite devastating outcomes of past RVF outbreaks, there are no licensed vaccines or antivirals available for humans. In the U.S., the handling of RVFV, which is a risk group 3 pathogen, requires biosafety level (BSL) 3 or 4 laboratory, whereas the possession, use, and transfer of RVFV are strictly under control of federal select agent program. A live-attenuated MP-12 vaccine strain is excluded from the select agent list and can be handled in BSL2 in the U.S., yet most other countries still require BSL3 for the handling of MP-12 strain. Nevertheless, the use of RVFV is required for basic and translational research to develop countermeasures against RVF. The reverse genetics is the technology to rescue infectious recombinant RNA viruses from cloned cDNA [6]. It allows manipulation of RNA virus genome, which has contributed to virology and vaccinology since the discovery. RVFV has also been rescued from cloned cDNA [7]. Recombinant MP-12 (rMP-12), including that expressing reporter gene, has been generated for the use in BSL2 for basic virology, antiviral screening and vaccine sciences. A development of reverse genetics system for other phleboviruses has been reported for Uukuniemi virus (UUKV) or Severe Fever with Thrombocytopenia Syndrome virus (SFTSV) [8–10]. UUKV and SFTSV are transmitted by ticks, and phylogenetically related to RVFV distantly. Recently, SFTSV was renamed as Huaiyangshan banyangvirus and reclassified into the genus *Banyangvirus*.

The order *Bunyavirales*, which consists of 12 families and 45 genera, contains at least 289 viral species with tripartite genomic RNA, comprising Large (L), Medium (M), and Small (S) segments [11]. The genus *Phlebovirus* within the family *Phenuiviridae* contains ten viral species (*Bujaru*, *Candiru*, *Chilibre*, *Frijoles*, *Punta Toro*, *Rift Valley fever*, *Salehabad*, *Sandfly fever Naples*, *Mukawa*, and *Uukuniemi phleboviruses*); meanwhile, many distinct phleboviruses that are phylogenetically close to those ten species remain unclassified. Many of those unassigned phleboviruses are cross-reactive to one of the ten phlebovirus species in serological assays, forming species complexes within the genus [12–16]. Arthropod vectors, including mosquitoes, sandflies, and ticks, play a major role in phleboviral transmission in nature and a number of the abovementioned viruses, specifically RVFV, Arumowot virus (AMTV), Odrenisrou virus, Icoaraci virus, and Itaporanga virus, are known to be transmitted via mosquitoes [17]. Geographically, RVFV, AMTV, and Odrenisrou virus are distributed in Africa, whereas the Icoaraci and Itaporanga viruses are present in South America.

In this study, we aimed to generate T7 RNA polymerase-driven reverse genetics system for AMTV, because AMTV is a mosquito-borne phlebovirus endemic to Africa, which is likely an excellent surrogate model to study virological similarity with pathogenic RVFV. AMTV remains unclassified, yet likely belongs to the Salehabad species complex [14]. It is widely distributed throughout the RVF-endemic region in Africa, where it has been isolated from *Culex antennatus*, *C*. *rubinotus*, *Mansonia uniformis*, *Tatera kempii* (gerbil), *Arbicanthis noloticus* (African grass rat), *Thamnomys macmillani* (wild rat), *Lemnyscomys striatus* (typical striped grass mouse), *Crocidura* sp. (shrew), and *Turdus libonyanus* (Kurrichane thrush) [18, 19]. Serological evidence of AMTV infection has also been reported in sheep in Burkina Faso and humans in Sudan, Egypt, and Somalia [20, 21], whereas no human or animal illnesses have been reported with AMTV. Experimental infection of sheep with 1 x 10^6^ PFU of AMTV resulted in transient fever in 50% of infected animals, but animals were asymptomatic without detectable viremia [22]. Similarly, experimental infection of Syrian hamsters with 10^5.4^ PFU of AMTV resulted in transient viremia with a peak at 3 days post infection (dpi) (10^7^ PFU/ml), but the infected animals did not succumb to the disease [23]. AMTV can also show an age-dependent pathogenicity in laboratory mice (i.e., death in suckling and weaning mice via intraperitoneal inoculation or intracerebral), according to the International Catalogue of Arbovirus by the Center for Disease Control and Prevention.

The nonstructural protein S (NSs protein) of RVFV is a major virulence factor, which promotes the post-translational degradation of dsRNA-dependent protein kinase (PKR) and transcription factor (TF) IIH subunit p62 [24–29], and interacts with TFIIH p44 subunit and sin3A associated protein 30 [30, 31]. RVFV-infected cells undergo general transcriptional suppression due to the failure of TFIIH assembly, whereas IFN-β gene up-regulation can be inhibited due to a failure of promoter activation at transcriptional level [32]. Pathogenic SFTSV encodes NSs proteins, which inhibit IFN-β gene up-regulation and the signaling pathway via forming inclusion bodies sequestering IFN regulatory factor 3 (IRF3) or cellular signal transducer and activator of transcription 2 (STAT2) [33–35]. NSs protein of nonpathogenic UUKV was dispensable for the replication in type-I IFN competent A549 cells, yet the replication was increased up to 10-fold with the expression of NSs protein via weakly inhibiting type-I IFNs [9]. We hypothesized that AMTV NSs protein is dispensable for efficient replication of recombinant AMTV (rAMTV) in type-I IFN-incompetent cells, whereas AMTV NSs proteins support robust viral replication in type-I IFN-competent cells. This study shows an establishment of the first reverse genetics for AMTV, a mosquito-borne phlebovirus distinct from RVFV, and the characterization of recombinant AMTV with and without NSs genes in human and murine cells.

## Methods

### Media, cells, and viruses

African green monkey kidney epithelial (Vero, ATCC CCL-81; Vero E6, ATCC CRL-1586), human lung diploid (MRC-5, ATCC CCL-171), human lung epithelial carcinoma (A549, ATCC CCL-185), human uterus endometrium (Hec1B, ATCC HTB-113), mouse hepatoma (Hepa1-6, ATCC CRL-1830), and mouse embryonic fibroblast (MEF-BL/6-1, ATCC SCRC-1008) cells were maintained at 37°C with 5% CO_2_ in Dulbecco’s modified minimum essential medium containing 10% fetal bovine serum (FBS), penicillin (100 U/ml), and streptomycin (100 μg/ml). Baby hamster kidney cells that stably expressed T7 RNA polymerase (BHK/T7-9 cells) [36] were maintained at 37°C with 5% CO_2_ in minimum essential medium alpha containing 10% FBS, penicillin (100 U/ml), streptomycin (100 μg/ml), and hygromycin B (600 μg/ml). All cells used in this study were verified to be mycoplasma free at the University of Texas Medical Branch at Galveston (UTMB) Tissue Culture Core Facility, and the identities of MRC-5, A549, and Hec1B cells were authenticated by Short Tandem Repeat analysis (UTMB Molecular Genomics Core Facility). AMTV Ar 1286–64 strain (wt AMTV: originally isolated from *Culex antennatus* in Sudan in 1963, and passaged in suckling mice twice and in Vero cells once) was kindly provided by Dr. Robert B. Tesh of the University of Texas Medical Branch at Galveston (UTMB) via the World Reference Center for Emerging Viruses and Arboviruses. AMTV was plaque-cloned once and then amplified three times in Vero cells before experimental use. Rescued rAMTV, rAMTV lacking the NSs gene (rAMTVΔNSs), or rAMTV encoding green fluorescent protein (GFP) gene in place of AMTV NSs gene (rAMTV-GFP) were also amplified once in Vero cells after their recovery using BHK/T7-9 cells. A parental rMP-12 or rMP-12 encoding an in-frame 69% deletion of the NSs gene (rMP12-ΔNSs16/198, alternative name: rMP12-C13type) has been previously described [7]. The rMP-12 encoding GFP gene in place of MP-12 NSs gene (rMP12-GFP), or that encoding AMTV NSs gene in place of MP-12 NSs gene (rMP12-AMTVNSs) were amplified once in Vero cells after their recovery using BHK/T7-9 cells. All stock viruses were sequenced (Sanger sequencing), titrated via plaque assay using Vero E6 cells, and then used for subsequent experiments.

### Plasmids

Plasmids encoding positive-sense L-, M-, or S-segments of AMTV prototype strain, Ar 1286–64 (GenBank accession numbers; MF593931—MF593933), flanked by an upstream T7 promoter (non-templated 2G in T7 promoter) and a downstream hepatitis delta virus ribozyme, were prepared using a custom DNA synthesis service (GenScript Inc.), and designated pProT7-AMTV-S(+), pProT7-AMTV-M(+), or pProT7-AMTV-L(+), respectively. Plasmids expressing N, L, or Gn/Gc proteins of AMTV were also prepared by subcloning the open reading frame (ORF) of each gene into pCAGGS or pT7-IRES plasmids, and designated pCAGGS-AMTV-N, pT7-IRES-AMTV-L, or pCAGGS-AMTV-G, respectively [37]. The pProT7-AMTV-S(+)-ΔNSs plasmid, which encodes AMTV S-segment with a 97% in-frame truncation (aa. 9 to aa. 249) within the NSs ORF (267 amino acids), was constructed via site-directed mutagenesis, whereas the pProT7-AMTV-S(+)-GFP or pProT7-AMTV-S(+)-rLuc plasmids were generated by replacing the NSs ORF with GFP or rLuc ORF, respectively. The pSPT18-AMTV-S plasmid was constructed by subcloning a partial AMTV N ORF into the pSPT18 plasmid (Sigma-Aldrich).

### Rescue of recombinant viruses

For the rescue of rAMTV from cloned cDNA, subconfluent monolayers of BHK/T7-9 cells in 60-mm dishes were co-transfected with 1.5 μg of pProT7-AMTV-S(+), 1.5 μg of pProT7-AMTV-M(+), 1.5 μg of pProT7-AMTV-L(+), 2.8 μg of pCAGGS-AMTV-N, 0.35 μg of pT7-IRES-AMTV-L, or 1.4 μg of pCAGGS-AMTV-G, using TransIT-293 transfection reagent (Mirus Bio LLC.). At 24 hours post-transfection, the culture medium was replaced with fresh medium. Five days after transfection, the culture supernatants were transferred into Vero cells for further viral amplification. For the rescue of rAMTV-ΔNSs, rAMTV-GFP, or rAMTV-rLuc, 1.5 μg of the pProT7-AMTV-S(+)-ΔNSs, pProT7-AMTV-S(+)-GFP, or pProT7-AMTV-S(+)-rLuc plasmid were used, respectively, in place of the pProT7-AMTV-S(+) plasmid. Rescue of rMP12-GFP, rMP12-rLuc, or rMP12-AMTVNSs was conducted using the same protocol described previously [7, 38].

### Viral replication kinetics study

The replication kinetics of rAMTV, or rAMTVΔNSs were tested in Vero, MRC-5, A549, Hepa1-6, and MEF cells (MOI of 0.01), whereas the replication kinetics of rMP-12, rMP12-ΔNSs16/198, or rMP12-AMTVNSs were tested in MRC-5, or Hepa1-6 cells (MOI of 0.01). After one hour incubation at 37°C, cells were washed three times with PBS, and then further incubated at 37°C. Culture supernatants were harvested at 1, 24, 48, 72, and 96 hours post infection (hpi), and viral titers were measured by plaque assay. The replication kinetics of rAMTV-GFP or rMP12-GFP were also tested in Vero cells, and titers were measured by focus-forming assay detecting the GFP signals of infected cells. Growth curves were calculated using means and standard deviations from three independent experiments.

### Western blot analysis

For the detection of AMTV protein synthesis, Vero cells were mock-infected or infected with wt AMTV, rAMTV, or rAMTVΔNS at 2 MOI, and lysates were collected at 8 or 16 hpi. For the detection of AMTV NSs protein, MEF, Hepa1-6, MRC-5, or A549 cells were mock-infected or infected with rMP12-AMTVNSs at 3 MOI, and lysates were collected at 16 or 24 hpi. For the analysis of IRF3 phosphorylation, Hepa1-6 cells were mock-infected or infected with rMP12-ΔNSs16/198 or rMP12-AMTVNSs at 10 MOI, and lysates were collected at 8 hpi. Western blot analysis was performed using Immobilon P polyvinylidene fluoride membranes (Millipore), as described previously [37]. The following primary antibodies were used: anti-AMTV N peptide rabbit polyclonal antibody (peptide N-KLIIERGGNNWKEDAK-C, ProSci Inc.), anti-AMTV NSs rabbit polyclonal antibody (custom antibody production via Proteintech group, using recombinant whole AMTV NSs with a Nus-tag and His-tag prepared via the UTMB Protein Chemistry Core), anti-RVFV hyper-immune mouse ascetic fluid (kindly provided by Dr. R. B. Tesh at UTMB), anti-IRF3 rabbit monoclonal antibody (Cell Signaling Technologies), anti-phosphorylated IRF3 Ser396 (Cell Signaling Technologies), anti-phosphorylated IRF3 Ser386 (Sigma Aldrich), anti-actin goat polyclonal antibody, anti-β-actin goat polyclonal antibody, anti- glyceraldehyde 3-phosphate dehydrogenase (GAPDH) goat polyclonal antibody (Santa Cruz Biotechnology). The following secondary antibodies were used: goat anti-mouse IgG-horseradish peroxidase (HRP) antibody (Santa Cruz Biotechnology), goat anti-rabbit IgG-HRP antibody (Invitrogen), or donkey anti-goat IgG-HRP antibody (Santa Cruz Biotechnology). For the inhibition of proteasome, lysosome, or cellular transcription, cells were treated with MG132 (5, 10, or 20 μM), chloroquine (10, 50, or 100 μM), or actinomycin D (1, 3, or 5 μg/ml) (Sigma-Aldrich), respectively, starting at 1 hpi.

### Analysis of the cell number expressing GFP

Monolayers of Vero, MRC-5, A549, Hec1B, Hepa1-6, or MEF cells in 12-well plate were incubated with rMP12-GFP or rAMTV-GFP (input: 7.0 x 10^5^ PFU/well) at 37°C for 1 hr. After removal of inocula, cells were further incubated with fresh media at 37°C. At 8 hpi, cells were trypsinized and resuspended into 400μl media. The numbers of total cells and GFP-expressing cells were measured by using Countess II FL Automated Cell Counter with EVOS LED Cube GFP (Thermo Fisher Scientific). The percentages of GFP-positive cells in the number of theoretically infected cells based on viral input were calculated per cell type.

### Inhibition of rAMTV-rLuc or rMP12-rLuc by broadly-active antivirals

Vero cells were mock-infected or infected with rAMTV-rLuc or rMP12-rLuc at 2 MOI. After 1 hour adsorption at 37°, cells were treated with 1, 10, or 100 μg/ml of favipiravir (Selleck Chemicals) or ribavirin (Sigma Aldrich). As a control, cells were treated with DMSO at the amount equivalent to that used for 100 μg/ml of favipiravir or ribavirin. Culture supernatants were collected at 1 hpi and 24 hpi, and virus titers (PFU/ml) were measured by plaque assay. Cell lysates were harvested at 24 hpi, and rLuc activities were measured by Renilla Luciferase Assay System (Promega), according to the manufacturer’s instructions. The relative rLuc activities were normalized to the protein concentrations of lysates, which were measured by Qubit Protein Assay Kit (Thermo Fisher Scientific).

### Northern blot analysis

MRC-5 cells were mock-infected or infected with wt AMTV, rAMTV, or rAMTVΔNSs at 2 MOI, and total RNA was extracted using the TRIzol reagent (Invitrogen) at 8 or 16 hpi. Approximately 100 ng of denatured RNA were separated on 1% denaturing agarose-formaldehyde gels and transferred onto a nylon membrane (Roche Applied Science). Northern blot analysis was performed, as described previously [39], with strand-specific RNA probes to detect antiviral-sense AMTV S-segment RNA within the N ORF. A RNA probe for the AMTV S-segment was newly generated from pSPT18-AMTV-S plasmid via *in vitro* transcription, using the DIG RNA Labeling Kit (Sigma-Aldrich) according to the manufacturer’s instructions. Glyceraldehyde 3-phosphate dehydrogenase (GAPDH) mRNA was also detected to visualize the level of cellular RNA.

### Droplet digital PCR

MRC-5 or Hepa1-6 cells were mock-infected or infected with 2 MOI of rAMTV or rAMTVΔNSs, or 10 MOI of rMP12-AMTVNSs, rMP-12, or rMP12-ΔNSs16/198. Total RNA was collected at 4, 8, or 16 hpi, and the copy numbers of human or murine IFN-β mRNA were analyzed by droplet digital PCR (ddPCR) using the QX100 droplet generator and reader (Bio-Rad Laboratories), as described previously [40]. Total RNA was extracted using the RNeasy mini kit (Qiagen), according to the manufacturer’s instructions. The concentration of extracted RNA was measured with a Qubit 2.0 Fluorometer (Thermo Fisher Scientific), and first-strand complementary DNA (cDNA) was synthesized using iScript (Bio-Rad Laboratory). PCR reactions were prepared as follows: 250 nM of TaqMan probe, 900 nM of primer, ddPCR Supermix for Probes (Bio-Rad Laboratory), cDNA, and water (up to 25 μl). PCR cycling parameters included an initial denaturation step (95°C for 10 minutes), followed by 40 cycles of 94°C for 30 seconds, 60°C for 1 minute, and a final denaturation step of 98°C for 10 minutes.

For the measurement of human IFN-β mRNA copy number, ddPCR was conducted using a Taqman probe, 5’-(5’ Hexachloro-Fluorescein, HEX) CAA TTG AAT GGG AGG CTT GAA TAC (Black Hole Quencher 1, BHQ1)-3’; forward primer, 5’-TCA GTG TCA GAA GCT CCT GT-3’; and reverse primer, 5’-GTT CAT CCT GTC CTT GAG GC-3’. For the measurement of murine IFN-β mRNA copy number, a different Taqman probe, 5’-(HEX) TGG AGA TGA CGG AGA TGC AGA (BHQ1)-3’; forward primer, 5’-TAC AGG GCG GAC TTC AAG AT-3’; and reverse primer, 5’-TGG CAA AGG CAG TGT AAC TC-3’, were used. The number of droplets with positive and negative signals was measured using the QX100 droplet reader. Data analysis was performed using QuantaSoft Version 1.4 (Bio-Rad Laboratory).

### Statistical analysis

Statistical analyses were performed using GraphPad Prism 6.05 (GraphPad Software Inc.). For comparisons among groups of viral titers or RNA copy numbers, arithmetic means of log_10_ values were analyzed by unpaired *t*-test (two samples), or one-way ANOVA, followed by Tukey’s multiple comparison test (more than two samples).

### Ethics statement

The RVFV MP-12 strain, rMP-12 strains, AMTV, and rAMTV strains, as well as recombinant DNA, were used upon approval of the Notification of Use by the Institutional Biosafety Committee at UTMB.

## Results

### Rescue of infectious AMTV from cloned cDNA

**Fig 1:**
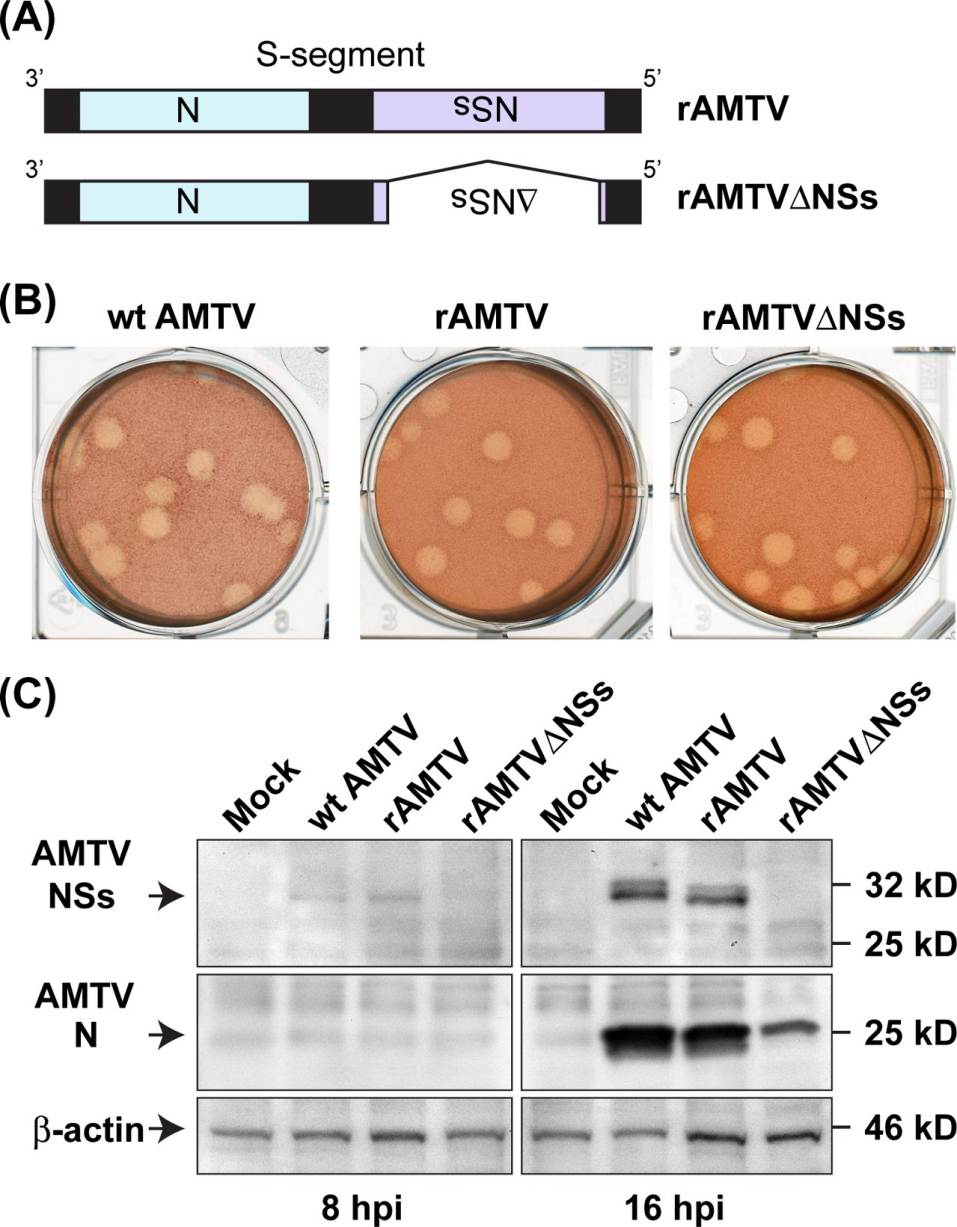
Rescue of infectious rAMTV from cloned cDNA. **(A)** Schematic representations of genomic S-segment RNAs of recombinant AMTV (rAMTV) and rAMTVΔNSs. **(B)** Plaques of wild-type (wt) AMTV, rAMTV and rAMTVΔNSs in Vero E6 cells at 4 days post infection under 0.6% noble agar. Cells were stained with neutral red for 16 hours. **(C)** Vero cells were mock-infected or infected with wt AMTV, rAMTV, or rAMTVΔNSs at a MOI of 2. Cell lysates at 8 and 16 hours post infection were analyzed by Western blot using anti-AMTV NSs antibody, anti-AMTV N antibody, and anti-β-actin antibody.

The T7 RNA polymerase-driven reverse genetics system, which has previously been used for the rescue of RVFV [7], was applied to rescue rAMTV strains. BHK/T7-9 cells were transfected with six plasmids expressing full-length genomic RNA (AMTV L-, M-, and S-segments) and structural viral proteins (AMTV N, L, and GnGc). At 5 days post transfection, culture supernatants were further amplified in Vero cells. Schematic representations of the S-segments of rAMTV and rAMTVΔNSs are shown in **Fig 1A**. Those AMTV strains were rescued successfully and showed a similar plaque morphology with wt AMTV in Vero E6 cells (**Fig 1B**). To confirm the expression of AMTV proteins, Vero cells were mock-infected or infected with wt AMTV, rAMTV, or rAMTVΔNSs (2 MOI). Western blot analysis using anti-AMTV-N antibody or anti-AMTV-NSs antibody showed that AMTV N protein accumulated in Vero cells infected with wt AMTV, rAMTV, or rAMTVΔNSs, whereas AMTV NSs protein was only detectable in cells infected with wt AMTV or rAMTV, not those with rAMTVΔNSs (**Fig 1C**).

### Characterization of rAMTV-GFP replication

**Fig 2:**
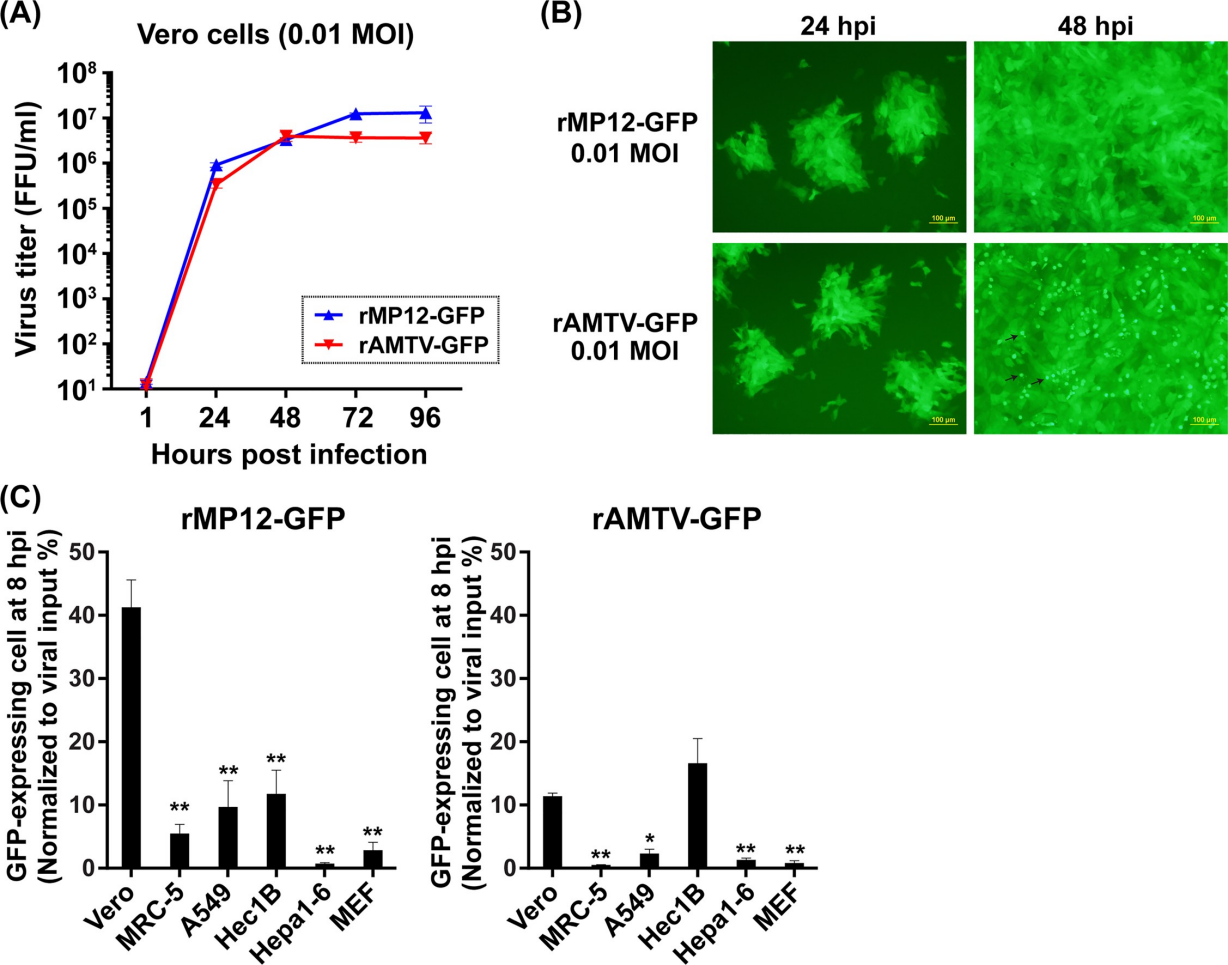
Characterization of rAMTV-GFP. **(A)** Replication kinetics of rMP12-GFP and rAMTV-GFP were analyzed in Vero cells at 0.01 MOI. **(B)** GFP expression of infected Vero cells (0.01 MOI) were detected at 24 and 48 hpi. Cells showing rounding are labeled in arrows. **(C)** After the infection with rMP12-GFP or rAMTV-GFP (7 x 10^5^ PFU input per well in 12-well plate), the number of GFP-expressing cells was measured at 8 hpi, and normalized to 7 x 10^5^ (100%). The means plus standard errors of triplicate experiments were shown in a graph. Arithmetic means of log_10_ values were analyzed via one-way ANOVA, followed by Tukey’s multiple comparison test. Asterisks represent statistical differences from the values of Vero cells (*p < 0.05, **p < 0.01).

Previous study showed that recombinant RVFV can express reporter gene from NSs gene [7]. Since the expression of GFP from AMTV will enable real-time detection of virus-infected cells without plaque assay or immunofluorescent assay, we next aimed to generate rAMTV expressing GFP, in place of NSs gene (rAMTV-GFP). Infectious rAMTV-GFP could be rescued from cloned cDNA successfully, and replicated as efficiently as recombinant RVFV MP-12 strain expressing GFP (rMP12-GFP), in Vero cells (**Fig 2A**). We used Vero cells (African green monkey kidney cells), because this cell line is susceptible to most phleboviruses due to lack the IFN-alpha/beta genes, and commonly used for virus amplification [41, 42]. The GFP expression pattern of rAMTV-GFP-infected Vero cells was indistinguishable from that of rMP12-GFP-infected Vero cells, whereas rAMTV-GFP infection led to detectable cytopathic effect at 48 hpi (**Fig 2B**). Since little is known about the infectivity of AMTV in culture cells, we next measured the number of GFP-expressing cells using several different culture cells derived from human or mice. Based on known susceptibility to RVFV MP-12 strain [43–46], we used three different human cells: MRC-5 cells (human lung diploid cells: type-I IFN-competent), A549 cells (human lung adenocarcinoma cell line: type-I IFN-competent), and Hec1B cells (human endometrial adenocarcinoma cell line: deficient in the early IFN signaling pathway via IFN-AR1 [47, 48]), and two different mouse cells: MEF cells (mouse embryonic fibroblast cells: type-I IFN-competent) and Hepa1-6 cells (murine hepatoma cell line: type-I IFN-competent). The number of GFP-expressing cells was measured and normalized to that of theoretically infected cells (100%) (**Fig 2C**); the GFP signals derived from rAMTV-GFP were seen in 11.4%, 16.6%, 0.5%, 2.3%, 1.3%, or 0.8% of Vero, Hec1B, MRC-5, A549, Hepa1-6, or MEF cells, respectively, whereas those from rMP12-GFP were seen in 41.3%, 11.8%, 5.5%, 9.7%, 0.7%, or 2.9%, respectively. The result indicated that Vero and Hec1B cells support the replication of rAMTV-GFP better than did MRC-5, A549, Hepa1-6, or MEF cells. Since rAMTV-GFP lacks NSs gene, it was possible that the replication of rAMTV-GFP was more restricted in type-I IFN-competent MRC-5, A549, Hepa1-6, or MEF cells than in type-I IFN-incompetent Vero or Hec1B cells.

### Generation of rAMTV-rLuc as a surrogate for rMP12-rLuc in antiviral screening assays

**Fig 3:**
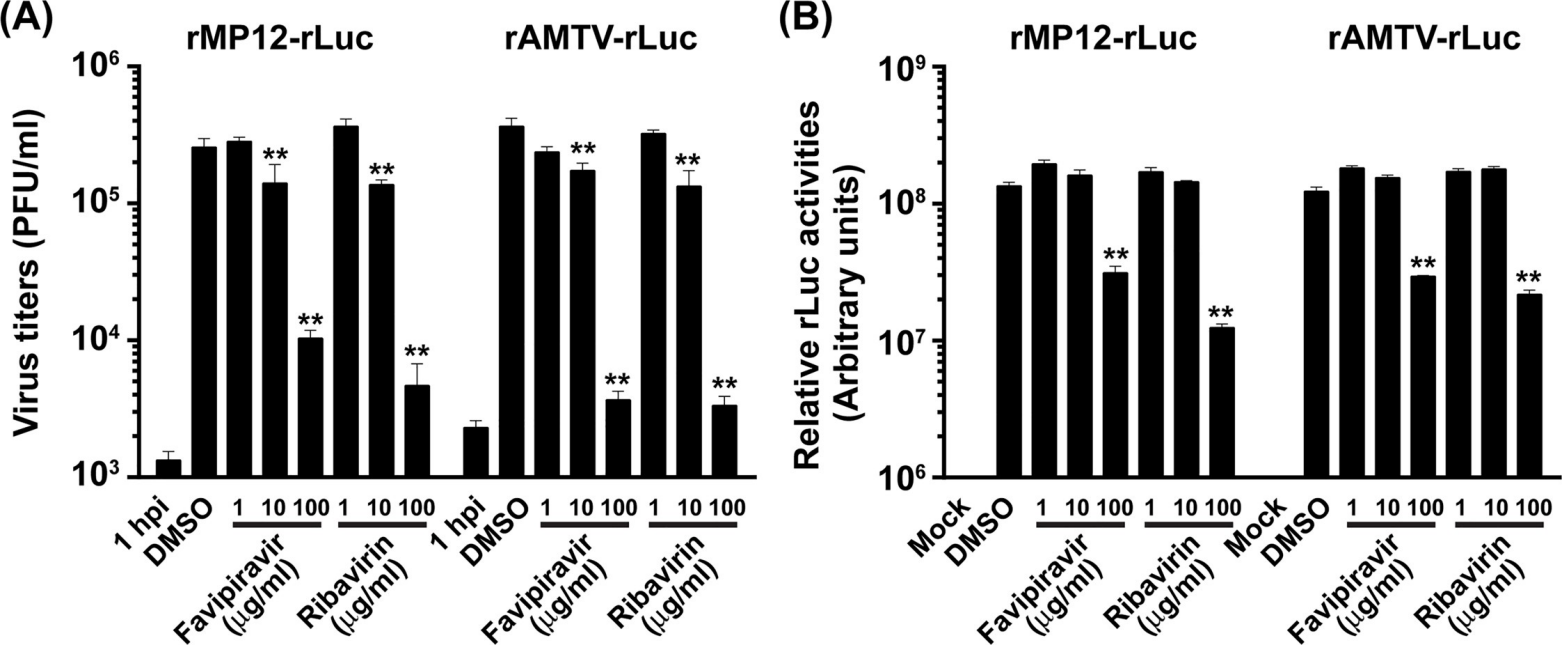
Susceptibility of rAMTV-rLuc and rMP12-rLuc to favipiravir or ribavirin in Vero cells. Vero cells were mock-infected or infected with rAMTV-rLuc or rMP12-rLuc at 2 MOI. After 1 hour adsorption at 37°C, cells were treated with 1, 10, or 100 μg/ml of favipiravir or ribavirin. As a control, cells were treated with DMSO at the amount equivalent to that used for 100 μg/ml of favipiravir or ribavirin. **(A)** Culture supernatants were collected at 1 hpi and 24 hpi, and virus titers (PFU/ml) were measured by plaque assay. **(B)** Cell lysates were harvested at 24 hpi, and rLuc activities were measured by luciferase assay. The relative rLuc activities were shown as the rLuc values normalized to the protein concentration of lysates. The means plus standard errors of triplicate experiments were shown in a graph. Arithmetic means of log_10_ values were analyzed via one-way ANOVA, followed by Tukey’s multiple comparison test. Asterisks represent statistical differences from the values of Vero cells (*p < 0.05, **p < 0.01).

The rMP12-rLuc, which expresses *Renilla* luciferase (rLuc) in place of NSs protein, has been used for antiviral screening in past studies [49–52]. Although the rMP12-rLuc is not pathogenic in animals and humans, works in BSL-3 facility will be required to handle MP-12 strain and the variants in most countries other than the U.S. Accordingly, surrogate viruses for rMP12-rLuc will likely be useful for antiviral screening in many countries. The median effective concentrations (EC_50_) of broad-spectrum antivirals, favipiravir (T-705, 6-fluoro-3-hydroxy-2-pyrazinecarboxamide) or ribavirin, for a Vero cell-based virus yield reduction assay against bunyaviruses were reported to be 5–30 μg/ml, or 13–42 μg/ml, respectively [53]. We generated the rAMTV-rLuc, which expresses rLuc in placed of NSs protein, by using the reverse genetics system, and tested the susceptibility to favipiravir or ribavirin. As shown in **Fig 3A**, Vero cells were mock-infected or infected with rAMTV-rLuc or rMP12-rLuc, and treated with 1, 10, or 100 μg/ml of favipiravir or ribavirin. With the treatment of 10 or 100 μg/ml of favipiravir, rAMTV-rLuc titers were reduced to 48 or 1%, and rMP12-rLuc titers were reduced to 56 or 4%, respectively. Similarly, with the treatment of 10 or 100 μg/ml of ribavirin, rAMTV-rLuc titers were reduced to 37 or 0.9%, and rMP12-rLuc titers were reduced to 53 or 1.8%, respectively. Different from infectious virus titers, the rLuc activities showed significant reduction at 100 μg/ml, but not 10 μg/ml, of favipiravir or ribavirin treatment (**Fig 3B**). The rLuc activities of rAMTV-rLuc or rMP12-rLuc at 100 μg/ml of favipiravir were 24% or 23%, respectively, whereas those of rAMTV-rLuc or rMP12-rLuc at 100 μg/ml of ribavirin were 18% or 9%, respectively. The results showed that the rAMTV-rLuc and rMP12-rLuc are similarly susceptible to favipiravir or ribavirin, and the rLuc activities derived from rAMTV-rLuc or rMP12-rLuc can be useful for rapid screening of efficacious antivirals without performing time-consuming plaque assays.

### Replication of rAMTV in type-I IFN competent cells

**Fig 4:**
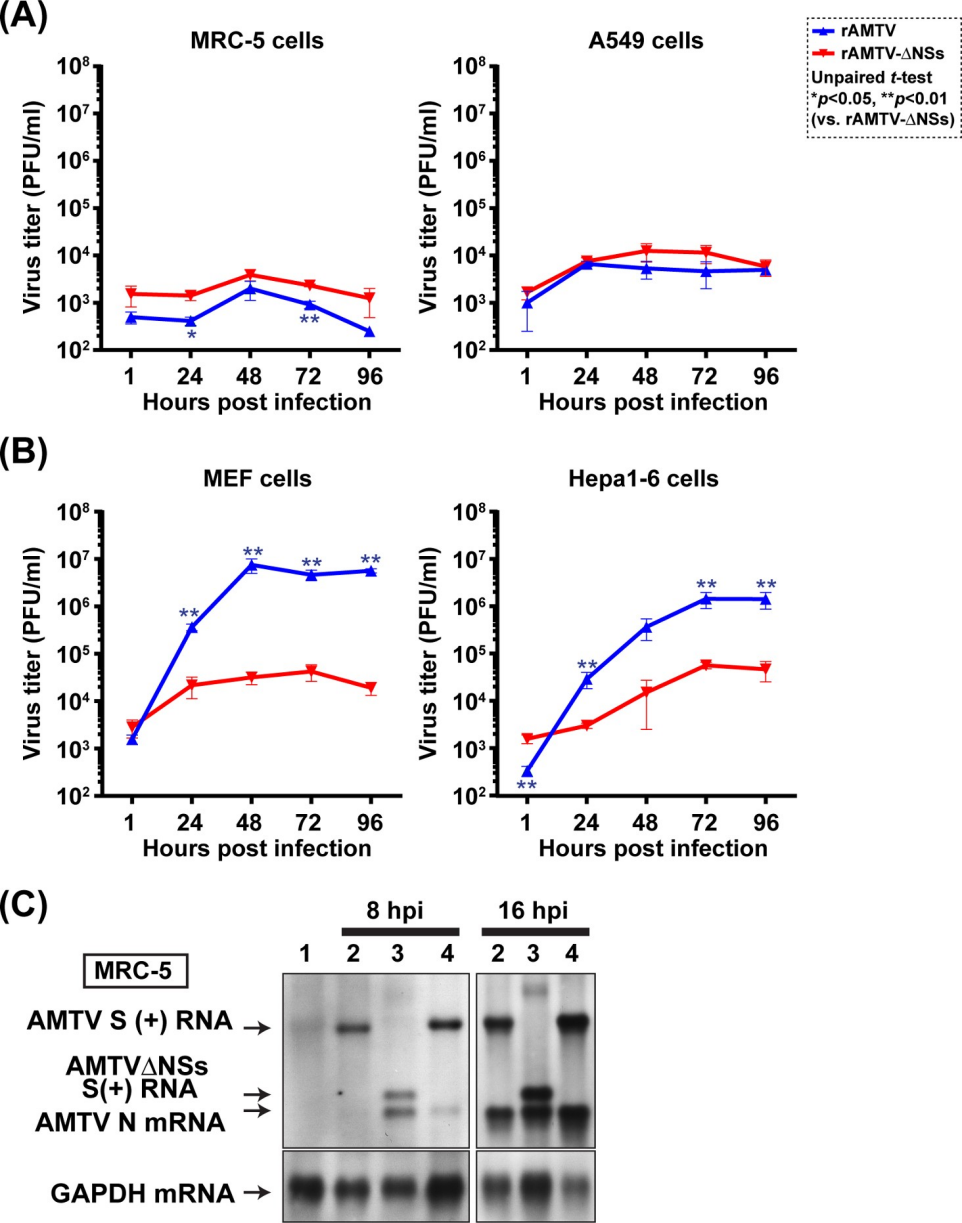
Replication kinetics of rAMTV and rAMTVΔNSs in type-I IFN competent cells. Replication kinetics of rAMTV or rAMTVΔNSs in **(A)** MRC-5, A549, or **(B)** MEF or Hepa1-6 cells were shown. Cells were infected with rAMTV or rAMTVΔNSs at 0.01 MOI. Experiments were performed in triplicate, and means ± standard errors are shown in graphs. Arithmetic means of log_10_ values were analyzed via unpaired *t*-test. Asterisks represent statistical differences among viral titers at corresponding time points (*p < 0.05, **p < 0.01). **(C)** MRC-5 cells were mock-infected (lane 1), or infected with wild-type (wt) AMTV (lane 2), rAMTVΔNSs (lane 3), or rAMTV (lane 4) at 2 MOI. Total RNA was extracted at 8 and 16 hpi, and Northern blot was performed using a RNA probe specific to the positive-sense AMTV S-segment (N open-reading frame) or glyceraldehyde 3-phosphate dehydrogenase (GAPDH) mRNA.

We next tested whether rAMTV expressing NSs protein can efficiently replicate in type-I IFN competent cells. MRC-5, A549, Hepa1-6, or MEF cells were infected with rAMTV or rAMTV-ΔNSs at MOI 0.01. Unexpectedly, neither rAMTV nor rAMTV-ΔNSs replicated efficiently in MRC-5 or A549 cells; i.e., peak titers of rAMTV were 2.0 x 10^3^ or 6.6 x 10^3^ PFU/ml in MRC-5 or A549 cells, respectively (**Fig 4A**). In contrast, rAMTV replicated more efficiently than rAMTV-ΔNSs in MEF or Hepa1-6 cells; i.e., the peak titers of rAMTV were 7.5 x 10^6^ PFU/ml (236 fold higher than rAMTV-ΔNSs titer) or 1.4 x 10^6^ PFU/ml (25 fold higher than rAMTV-ΔNSs titer) in MEF or Hepa1-6 cells, respectively (**Fig 4B**). The result indicated that AMTV NSs supports the replication of AMTV in type-I IFN-competent MEF or Hepa1-6 cells. In contrast, AMTV NSs protein apparently did not function in MRC-5 or A549 cells. Using Northern blot, the accumulation of AMTV S-segment RNA in MRC-5 cells was also analyzed (**Fig 4C**). Although positive-sense AMTV S-segment RNA was detected in all infected samples at 8 hpi, AMTV N mRNA was poorly detected in MRC-5 cells infected with wt AMTV or rAMTV at 8 hpi, and more detectable at 16 hpi, confirming that AMTV RNA synthesis occurs in MRC-5 cells.

### Expression of AMTV NSs from rMP-12 backbone

**Fig 5:**
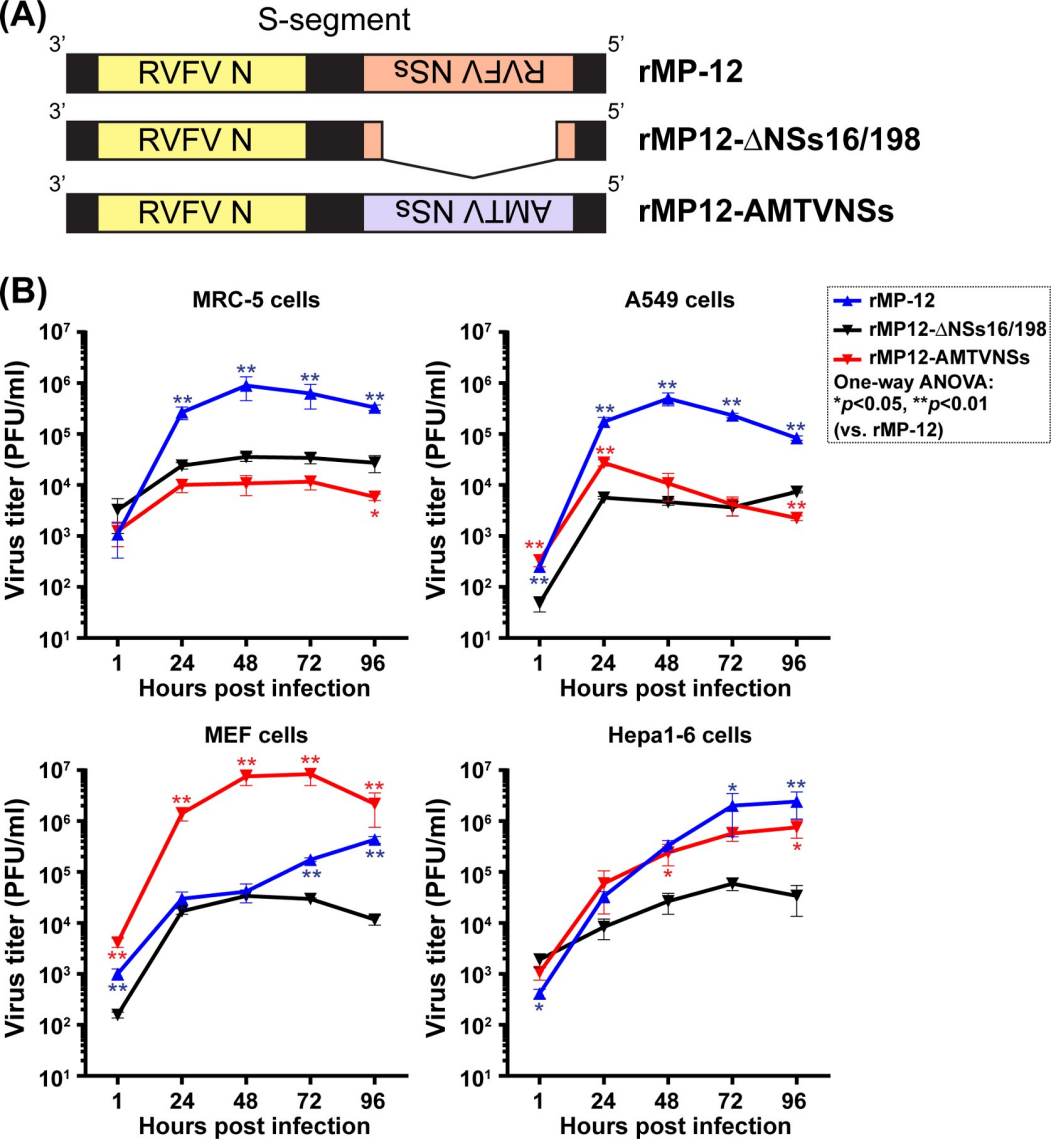
Expression of AMTV NSs protein from rMP-12 backbone. Recombinant MP-12 (rMP-12) encoding AMTV NSs gene in place of MP-12 NSs gene (rMP12-AMTVNSs) was generated. **(A)** The schematics of the S-segment of parental rMP-12, rMP12-ΔNSs16/198, and rMP12-AMTVNSs, **(B)** Replication kinetics of rMP-12, rMP12-ΔNSs16/198, or rMP12-AMTVNSs in MRC-5, A549, MEF, or Hepa1-6 cells. Cells were infected with rMP-12, rMP12-ΔNSs16/198, or rMP12-AMTVNSs at 0.01 MOI. Experiments were performed in triplicate, and means ± standard errors are shown in graphs. Arithmetic means of log_10_ values were analyzed via one-way ANOVA, followed by Tukey’s multiple comparison test. Asterisks represent statistical differences from the values of rMP12-ΔNSs16/198 (*p < 0.05, **p < 0.01).

To further analyze the role of AMTV NSs proteins, we next transferred AMTV NSs gene into rMP-12 S-segment, in place of MP-12 NSs gene (**Fig 5A**). MP-12 NSs protein can inhibit the activation of IFN-β promoter and thus rMP-12 can replicate efficiently in type-I IFN-competent cells. If AMTV NSs functions as an antagonist for type-I IFN, the AMTV NSs protein should support efficient replication of rMP-12 in type-I IFN-competent cells. As shown in **Fig 5B**, the replication of rMP12-AMTVNSs was overall not efficient, and similar to that rMP12-ΔNSs16/198 in MRC-5 and A549 cells, whereas the replication of rMP-12 was significantly more efficient than that of rMP12-ΔNSs16/198 in MRC-5 or A549 cells. In contrast, the rMP12-AMTVNSs and rMP-12 replicated more efficiently than rMP12-ΔNSs16/198 in Hepa1-6 cells or in MEF cells. The result confirmed that AMTV NSs proteins can support efficient rMP-12 replication in type-I IFN competent murine MEF or Hepa1-6 cells, but not in MRC-5 or A549 cells.

### Posttranslational degradation of AMTV NSs in human cells

**Fig 6:**
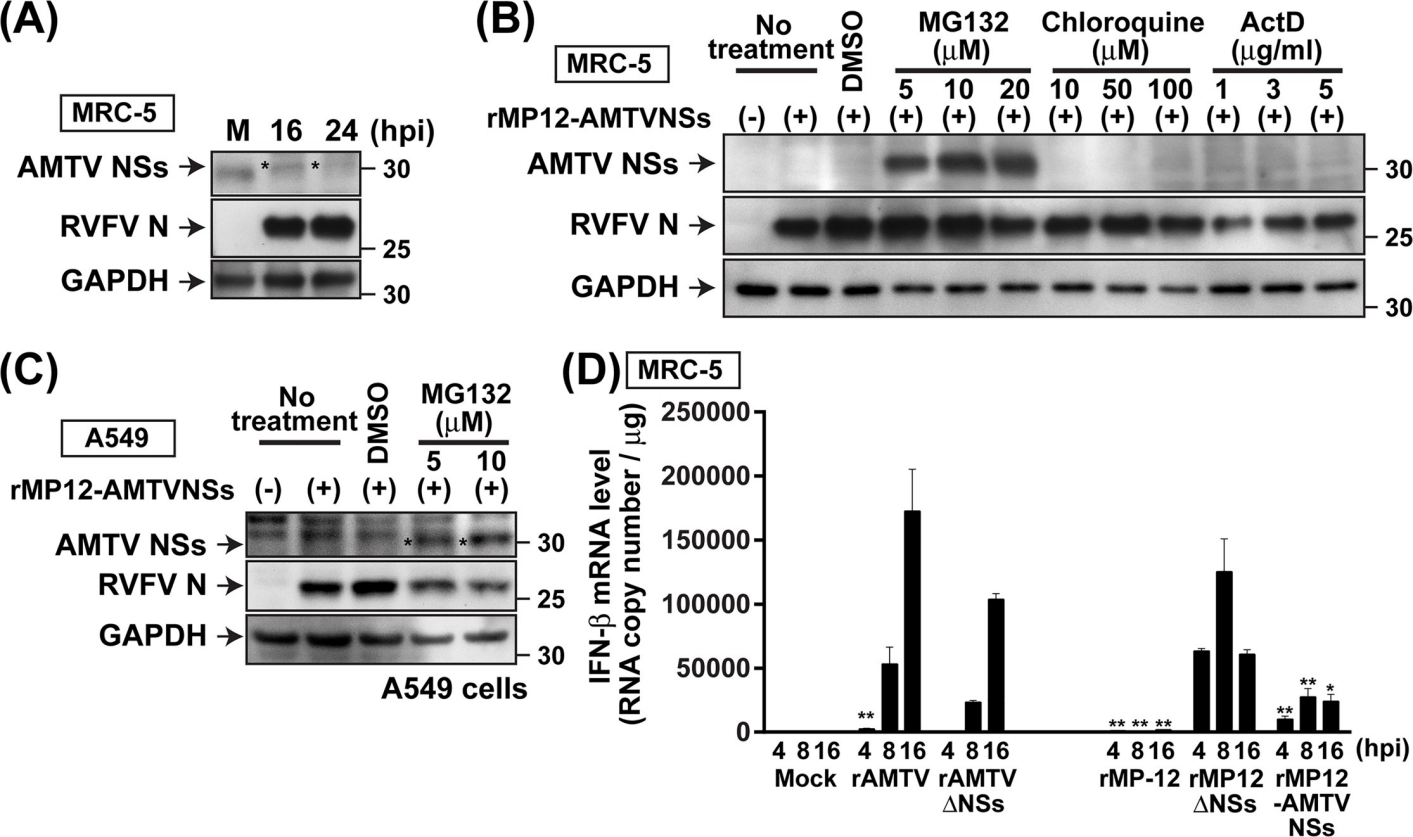
Posttranslational degradation of AMTV NSs proteins in human cells. **(A)** MRC-5 cells were mock-infected or infected with rMP12-AMTVNSs at 3 MOI. Cell lysates were collected at 16 or 24 hpi. Western blot was performed using anti-AMTV NSs antibody, anti-RVFV hyper-immune mouse ascetic fluid, or anti-GAPDH antibody. Asterisks indicate faint accumulations of AMTV NSs. **(B)** MRC-5 cells were mock-infected or infected with rMP12-AMTVNSs at 3 MOI. Cells were non-treated or immediately treated with DMSO, MG132, chloroquine, or actinomycin D (ActD) at indicated concentrations. Cell lysates were harvested at 16 hpi, and Western blot was performed using anti-AMTV NSs antibody, anti-RVFV hyper-immune mouse ascetic fluid, or anti-GAPDH antibody. **(C)** A549 cells were mock-infected or infected with rMP12-AMTVNSs at 3 MOI. Cells were non-treated or immediately treated with DMSO or MG132 at indicated concentrations. Cell lysates were collected at 16 hpi, and Western blot was performed using anti-AMTV NSs antibody, anti-RVFV hyper-immune mouse ascetic fluid, or anti-GAPDH antibody. Asterisks indicate accumulations of AMTV NSs. **(D)** MRC-5 cells were mock-infected, or infected with rAMTV or rAMTVΔNSs at 2 MOI, or rMP-12, rMP12-AMTVNSs or rMP12-ΔNSs16/198 (rMP12ΔNSs) at 10 MOI. Total RNA was extracted at 8 or 16 hpi, and droplet digital PCR analysis was performed using human IFN-β probe. The Y-axis represents the RNA copy number per μg of total RNA. Experiments were performed in triplicate, and means ± standard errors are shown in graphs. Arithmetic means of log_10_ values were analyzed via one-way ANOVA, followed by Tukey’s multiple comparison test. Asterisks represent statistical differences from the values of NSs-deletion mutants (rAMTVΔNSs or rMP12-ΔNSs16/198) at each time point (*p < 0.05, **p < 0.01).

To analyze the expression level of AMTV NSs proteins, MRC-5 were mock-infected or infected with rMP12-AMTVNSs at 3 MOI (**Fig 6A**). Although MP-12 N proteins could be detected abundantly in MRC-5 cells infected with rMP12-AMTVNSs at 16 and 24 hpi, there was little accumulation of AMTV NSs proteins. We hypothesized that the AMTV NSs proteins are posttranslationally degraded via proteasome pathway. Alternatively, AMTV NSs proteins might be degraded via lysosome, or NSs gene expression might be downregulated via cellular antiviral responses induced via viral replication. To test this, MRC-5 cells were mock-infected or infected with rMP12-AMTVNSs at 3 MOI. Cells were either non-treated, DMSO-treated, or treated with a proteasome inhibitor MG132 (5, 10, 20 μM), a lysosomal inhibitor, chloroquine (10, 50, 100 μM), or cellular transcriptional inhibitor, actinomycin D (1, 3, 5 μg/ml). Cells were then harvested at 16 hpi for Western blot analysis. As shown in **Fig 6B**, infected MRC-5 cells treated with MG132, but not with chloroquine or actinomycin D, stabilized AMTV NSs proteins. Similarly, AMTV NSs proteins were not detectable in mock-treated A549 cells, yet AMTV NSs protein was stabilized with MG132 (**Fig 6C**). The result showed that MRC-5 or A549 cells promote post-translational degradation of AMTV NSs proteins via proteasome. The level of IFN-β mRNA upregulation was analyzed by ddPCR using total RNA extracted from MRC-5 cells, which were mock-infected or infected with rAMTV, rAMTVΔNSs, rMP-12, rMP12-AMTVNSs, or rMP12-ΔNSs16/198 (**Fig 6D**). Analysis of copy numbers of human IFN-β mRNA showed that rAMTV infection induced 2,467, 53,130, or 172,613 copies of human IFN-β mRNA in MRC-5 cells at 4, 8, or 16 hpi, which was 11, 110, or 625 times more than that of mock-infected cells, respectively. Infection by rAMTVΔNSs also induced levels of human IFN-β mRNA similar to those of rAMTV-infected cells at 8 and 16 hpi. Similarly, rMP12-AMTVNSs induced 9,933, 27,383, or 24,007 copies of human IFN-β mRNA at 4, 8, or 16 hpi, which was 43, 57, or 87 times more than that of mock-infected cells, respectively. Infection by rMP12-ΔNSs16/198 induced 275, 259, or 220 times more human IFN-β mRNA than mock-infected controls at 4, 8, and 16 hpi, respectively, whereas an increase of human IFN-β mRNA induced by parental rMP-12 was within 6 times, compared to the mock-infected control. The result indicated that rapid degradation of AMTV NSs proteins dampens any biological roles of AMTV NSs proteins, if any, in those cell types derived from humans.

### Inhibition of murine IFN-β gene upregulation by AMTV NSs proteins

**Fig 7:**
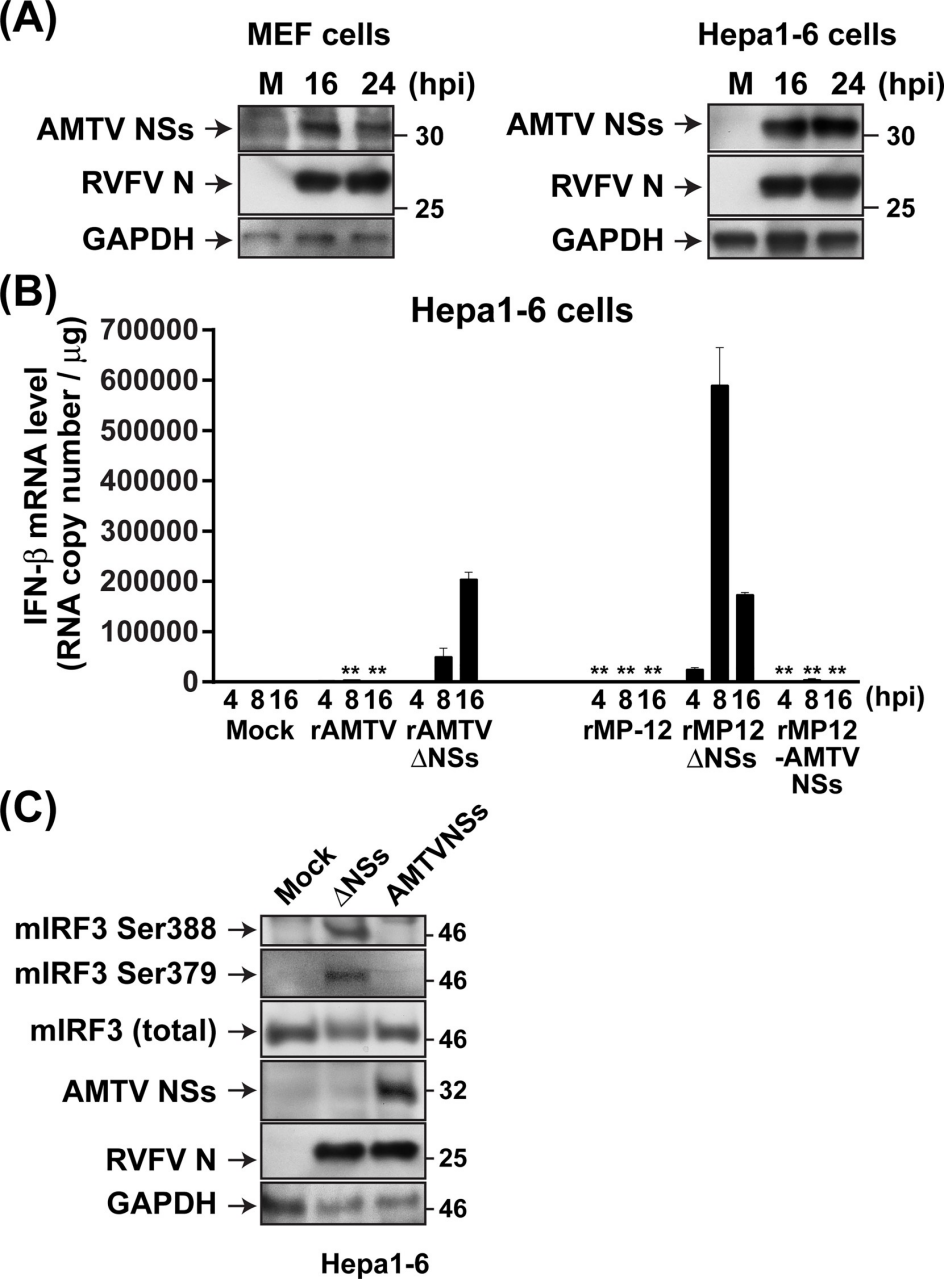
Inhibition of murine IFN-β gene upregulation by AMTV NSs proteins. **(A)** MEF or Hepa1-6 cells were mock-infected or infected with rMP12-AMTVNSs at 3 MOI. Cell lysates were collected at 16 or 24 hpi. Western blot was performed using anti-AMTV NSs antibody, anti-RVFV hyper-immune mouse ascetic fluid, or anti-GAPDH antibody. **(B)** Hepa1-6 cells were mock-infected, or infected with rAMTV or rAMTVΔNSs at 2 MOI, or rMP-12, rMP12-AMTVNSs or rMP12-ΔNSs16/198 (rMP12ΔNSs) at 10 MOI. Total RNA was extracted at 8 or 16 hpi, and droplet digital PCR analysis was performed using murine IFN-β probe. The Y-axis represents the RNA copy number per μg of total RNA. Experiments were performed in triplicate, and means ± standard errors are shown in graphs. Asterisks represent statistical differences from the values of NSs-deletion mutants (rAMTVΔNSs or rMP12-ΔNSs16/198) at each time point (*p < 0.05, **p < 0.01). **(C)** Hepa1-6 cells were mock-infected or infected with rMP12-ΔNSs16/198 (rMP12ΔNSs) or rMP12-AMTVNSs (AMTVNSs) at 10 MOI. Western blot analysis was performed using cell lysates at 8hpi with antibodies specific to IRF3 (total), phosphorylated IRF3 (Ser 386 or Ser 396), AMTV NSs, RVFV, or GAPDH. Murine IRF3 (mIRF3) phosphorylation sites corresponding to the Ser 386 and Ser 396 of human IRF3 are Ser 379 and Ser 388, respectively.

Next, MEF or Hepa1-6 cells were mock-infected or infected with rMP12-AMTVNSs at 3 MOI (**Fig 7A**). Different from MRC-5 or A549 cells, AMTV NSs proteins could be robustly accumulated in MEF or Hepa1-6 cells. Using total RNA extracted from Hepa1-6 cells, which were mock-infected or infected with rAMTV, rAMTVΔNSs, rMP-12, rMP12-AMTVNSs, or rMP12-ΔNSs16/198, RNA copy numbers of IFN-β mRNA were measured by using ddPCR. Analysis of copy numbers of murine IFN-β mRNA showed that rAMTV infection induced 1,967, 3,287, or 1554 copies per total RNA (μg) of murine IFN-β mRNA in Hepa1-6 cells at 4, 8, or 16 hpi, which was 1.5, 9.7, or 2.4 times more than that of mock-infected cells (**Fig 7B**). In contrast, rAMTVΔNSs infection induced 1,533, 50,100 or 204,094 copies of murine IFN-β mRNA at 4, 8 or 16 hpi, respectively, which were 1.2, 148 or 315 times more than those of mock-infected cells, respectively. Similarly, rMP12-ΔNSs16/198 induced murine IFN-β mRNA 18.5, 1,736, or 267 times more than that of mock-infected cells at 4, 8 or 16 hpi, respectively. Meanwhile, 1.4, 12, or 2.9 times or 1.3, 3.4, or 2.0 times more murine IFN-β mRNA were detected in Hepa1-6 cells infected with rMP12-AMTVNSs or parental MP-12 at 4, 8, or 16 hpi, respectively. These results showed that AMTV NSs expression can significantly inhibit the induction of murine IFN-β mRNA, and AMTV NSs protein functions as an antagonist of murine IFN-β mRNA upregulation. The IRF3 is one of the major transcription factors involved in inducing the activation of the IFN-β promoter. In humans, phosphorylation at serine (Ser) 385, 386, or 396 plays a major role in IRF3 activation via dimer formations [54–56]. The phosphorylation of IRF3 can be inhibited by SFTSV NSs, but not by RVFV NSs [32, 33]. We tested the phosphorylation of murine IRF3 at Ser 379 (corresponding to human IRF3 Ser 386) and Ser 388 (corresponding to human IRF3 Ser 396; **Fig 7C**) in Hepa1-6 cells mock-infected or infected with rMP12-ΔNSs16/198 or rMP12-AMTVNSs at 10 MOI. Phosphorylation of murine IRF3 at Ser 379 and 388 was detected in Hepa1-6 cells infected with rMP12-ΔNSs16/198, but not in those with rMP12-AMTVNSs. These results showed that murine IRF3 phosphorylation can be inhibited via the expression of AMTV NSs protein.

## Discussion

This study demonstrated the first rescue of infectious rAMTV (Sudan Ar 1286–64 strain) from cloned cDNA. The AMTV is a proposed member of the Salehabad virus species complex, which also include Salehabad virus (Iran), Arbia virus (Italy), Odrenisrou virus (Ivory Coast), Medjerda Valley virus (Tunisia), Adana virus (Turkey), Edrine virus (Turkey), Alcube virus (Portugal), Ponticelli virus (Italy), Olbia virus (France), and Adria virus (Albania) [14, 57–63]. We could successfully rescue rAMTV lacking the NSs gene (rAMTVΔNSs) or rAMTV expressing GFP (rAMTV-GFP) or rLuc (rAMTV-rLuc). The rAMTV-GFP could express GFP along with viral replication, which is useful to detect infected cells real-time. The rMP-12 variants expressing reporter gene (e.g., rMP12-rLuc) have been used for screening of antiviral compounds or host genes affecting viral replication [49–52]. The handling of MP-12 strain, however, still requires BSL-3 facility in many countries other than the U.S. AMTV is classified as a Risk Group 2 pathogen, and there are no known risk of human or animal diseases, which may be suitable for initial screening of broad-acting antivirals effective for both AMTV and RVFV worldwide. This study demonstrated that rAMTV-rLuc and rMP12-rLuc, which encodes rLuc gene in place of NSs gene in AMTV or MP-12 backbone, are similarly susceptible to broad-spectrum antivirals, favipiravir or ribavirin. The reduction of infectious virus titers were detectable at 10 μg/ml of favipiravir or ribavirin. Our study also showed a similar dose-dependent inhibition of rLuc activities from rAMTV-rLuc or rMP12-rLuc, by favipiravir or ribavirin treatment, yet the reduction of rLuc activities were detectable at 100 μg/ml, but not at 10 μg/ml. Since the transcription of NSs mRNA from antiviral-sense S-segment derived from virions can start immediately after infection [39], it is likely that rLuc mRNA could be accumulated in infected cells before favipiravir or ribavirin could inhibit viral RNA synthesis. Although further optimization of the timing of antiviral treatment may be required, rapid detection of reduced rLuc activities will be a useful method to screen antivirals, without performing time-consuming plaque assays for a large number of samples. The reverse genetics system for AMTV can generate not only NSs gene variants, but various other mutant strains, which can serve as an excellent tool to study basic virology (e.g., consensus gene elements functional among phleboviruses) and vaccinology (e.g., bunyavirus vector for expressing RVF GnGc antigens for vaccine use).

Post-translational degradation of AMTV NSs proteins occurred via proteasomes in MRC-5 and A549 cells. This phenomenon partly explains the lack of type-I IFN antagonist function of AMTV NSs observed in MRC-5 cells. It remains unknown whether similar degradation of AMTV NSs proteins occurs in authentic human tissues infected with AMTV. Nevertheless, it is likely that AMTV NSs serves as a substrate of ubiquitination for proteasomal degradation at least in MRC-5 and A549 cells. RVFV NSs proteins can promote the degradation of PKR and TFIIH p62, via forming unique E3 ligase complexes containing NSs proteins [24, 27–29, 64], whereas Toscana virus NSs protein promotes the degradation of PKR and RIG-I [65, 66]. The mechanism of AMTV NSs protein degradation in human cells should be further characterized in future studies.

AMTV efficiently replicated in type-I IFN-competent murine MEF and Hepa1-6 cells, whereas replication was significantly impaired by a lack of AMTV NSs protein expression. Moreover, rMP12-AMTVNSs, but not rMP12-ΔNSs16/198, robustly replicated in both MEF and Hepa1-6 cells. We also noted that the replication of rMP12-AMTVNSs was more efficient than that of parental rMP-12 in MEF cells. Accordingly, AMTV NSs proteins might function as a strong antagonist in the murine type-I IFN induction pathway. Consistent with this hypothesis, rAMTV and rMP12-AMTVNSs, which express AMTV NSs protein, inhibited the induction of murine IFN-β mRNA in infected Hepa1-6 cells. Moreover, the phosphorylation of murine IRF3 at Ser 378 and 388 was not detectable in Hepa1-6 cells infected with rMP12-AMTVNSs. These results indicate that activation of IRF3 or the upstream kinases (e.g., RIG-I) might be inhibited by AMTV NSs protein. Past isolations of AMTV from gerbils, African grass rats, wild rats, typical striped grass mice, and shrews [18] suggest that AMTV is transmitted among rodents and mosquitoes. It is thus likely that wild rodents can serve as natural reservoir harboring AMTV without exhibiting virulence. According to the International Catalogue of Arbovirus by the Center for Disease Control and Prevention, AMTV can cause lethal infection in suckling and weaning mice, but not in adult mice. The biological significance of AMTV NSs protein in murine cells remains unclear, because this study did not test cells derived from reservoir rodents. Antagonist function of IFN-β gene upregulation, if any, may allow transient viral replication in rodents, which support viral transmission via mosquito vectors. AMTV has also been isolated in *T*. *libonyanus* (Kurrichane thrush) in the Central African Republic, and the bird-derived AMTV strain (AnB7211d) showed 92.1% identity of NSs amino acid sequence with that of the Ar 1286–64 strain [19]. Future study is warranted for the differences between two different AMTV strains and the functions of NSs proteins in avian cells.
